# Comparison of modified versus conventional injection techniques of low-molecular-weight heparin in elderly

**DOI:** 10.12669/pjms.345.15466

**Published:** 2018

**Authors:** Wenzhen Geng, Ying Zhang, Juan Shi

**Affiliations:** 1 Wenzhen Geng, Internal Medicine Cardiovascular Department, Binzhou People’s Hospital, Shandong, 256610, China; 2 Ying Zhang, Pediatric Intensive Care Unit, Binzhou People’s Hospital, Shandong, 256610, China; 3 Juan Shi, Cardiothoracic Surgery Department, Binzhou People’s Hospital, Shandong, 256610, China

**Keywords:** Low-molecular-weight heparin, Modified injection, Subcutaneous bleeding

## Abstract

**Objective:**

To analyze the clinical values of modified injection of low-molecular-weight heparin in reducing subcutaneous bleeding and pain.

**Methods:**

Two hundred and sixty patients with cerebral infarction, acute myocardial infarction or pulmonary embolism who underwent subcutaneous injection of low-molecular weight heparin in the hospital between December 2015 and December 2016 were selected. They were randomly divided into a control group and a research group, 130 each. Patients in the control group were given conventional injection, while patients in the research group were given modified injection. The occurrence of subcutaneous bleeding and pain was observed and compared between the two groups.

**Results:**

The incidence of subcutaneous hemorrhage in the research group was 46.9%, significantly lower than 83.1% in the control group, and the difference had statistical significance (P<0.05). Twenty-six patients in the control group had severe pain, which was much more than 5 patients in the research group, and the difference was statistically significant (P<0.05). The number of cases of severe hemorrhage in the control group was significantly larger than that of the research group (31 vs. 3), and the difference was also statistically significant.

**Conclusion:**

Modified low-molecular weight heparin injection can effectively reduce the incidence of bleeding and pain, which is beneficial to the compliance and quality of life of elder patients.

## INTRODUCTION

Low-molecular-weight heparin, a novel anticoagulant developed in 1970s, has similar pharmacological effects with ordinary heparin sodium. It has advantages of high bioavailability, strong antithrombotic effect, long half-life period and low incidence of bleeding compared with ordinary heparin.^1^,^2^ Therefore it has been extensively applied to prevent and treat various thromboembolic diseases.^3^ Currently the main application mode of low molecular heparin is subcutaneous injection and intravascular injection is used in hematodialysis. Subcutaneous bleeding at injection sites is a common adverse reaction of subcutaneous injection of low molecular heparin, with an incidence of 90%.^4^ It is associated with blood clotting when blood enter subcutaneous tissue via the injury induced by needle head. Subcutaneous bleeding may be worsen if compression is not given timely; the diameter of bleeding area can be 3 to 4 cm; pain brought by bleeding can cause psychological burden, which can affect the medication compliance and drug absorption effect.^5^ Local clotting or bleeding is of higher risks among elder patients because they had special physiological characteristics such as aged body, weakened organic functions, slack skin and slow blood flow and fragile psychology.^6^,^7^

Few studies have addressed bleeding of elderly patients induced by injection of low molecular heparin. The authors modified injection of low-molecular heparin for elders, aiming to provide an evidence-based basis for clinical treatment and improve nursing quality and treatment compliance of elders.

## METHODS

### Research subjects

Two hundred and sixty patients with cerebral infarction, acute myocardial infarction or pulmonary embolism who needed injection of low-molecular-weight heparin in our hospital were selected between December 2015 and December 2016. Patients who had other vascular diseases or anaphylactic diseases or were transferred to another hospital during treatment were excluded. The inclusive criteria included 60 ~ 85 years, count of blood platelet and activated partial thromboplastin time within normal scopes, no large ecchymosis, induration and skin diseases on the abdomen, clear consciousness and normal language expression ability and listening. The included patients were randomly divided into a control group and a research group, 130 each. In the control group, there were 77 males and 53 females; they aged from 65~85 years (average (78.9±5.4) years). In the research group, there were 74 males and 56 females; they aged from 65 to 84 years (average (79.4±5.5) years). Age and gender were compared between the two groups, and no remarkable difference was observed (P>0.05); hence the results were comparable. All the included patients signed informed consent. This study has been approved by the ethics committee of our hospital.

### Methods

The same injection standard was used for all the patients. Each patient was injected with low-molecular-weight heparin using a prefabricated injector (Shenzhen SCIPROGEN Bio-pharmaceutical Co. Ltd., China), once every 12 h, 5000 U each time. All the patients were injected for 360 times. Patients in the control group were given conventional injection. After air was expelled from the needle tubing and local skin was disinfected, an operator tightened the skin at the injection site with his left hand and handled the injector with his right hand. With an angle of 30°~40° with the skin, the needle head was penetrated into subcutaneous tissue with the bevel upward. The needle was inserted into the skin for about 2/3 length of the needle stem. Drug was injected if no blood was observed after pumpback. The needle was pulled out after injection. Then the injection site was compressed with a cotton swab for 2 ~ 3 min.

The patients in the research group were given the modified injection. Before injection, the skin was swept in a conventional way. The injection site was given hot compression using a hot towel for 3~5 min and massaged. The skin was disinfected after hot compression. The operator pinched up the skin on the abdominal wall with a thumb and an index finger to form wrinkle. The needle was inserted vertically at the highest point of the wrinkle. The drug was rapidly poured for 10~15 s if no blood was observed after pumpback. Then the needle was pulled out. The skin was pinched up continuously for 3 to 5 min. The needle insertion site was not compressed.

### Assessment of curative effect

***Assessment of pain:***^8^ Pain was assessed as grade 0 ~ 10 according to numeric rating scale. No pain was grade 0, mild pain was from grade 1 to 3, moderate pain was from grade 4 to 6, and severe pain was from 7 to 10. Each patient selected a number to represent the degree of pain he felt.

***Assessment of subcutaneous hemorrhage:***^9^ whether there was induration and hematoncus or not at the injection site was observed at the 48th h after injection. The area of subcutaneous hemorrhage at the injection site was measured using a straightedge. Subcutaneous redness was evaluated as no bleeding; diameter of bleeding area smaller than 1 cm was evaluated as mild bleeding; diameter of bleeding area between 1 cm and 2 cm was evaluated as moderate bleeding; diameter of bleeding area larger than 2 cm was evaluated as severe bleeding.

### Statistical analysis

Data were analyzed using SPSS ver. 20.0. Ranked data were compared using Wilcoxon rank sum test. Enumeration data were processed by Chi-square test. Difference was thought as statistically significant if P<0.05.

## RESULTS

Comparison of bleeding incidence at the 48^th^ h after subcutaneous injection between the two groups: The incidence of bleeding of the research group was 46.9%, which was significantly lower than that of the control group (83.1%); the difference had statistical significance (P<0.05; Table-I).

**Table-I:** Comparison of incidence of subcutaneous bleeding between the two groups [n(%)].

Group	Number of cases of subcutaneous bleeding	Number of cases of non-subcutaneous bleeding
The research group	61 (46.9)	69 (53.1)
The control group	108 (83.1)	22 (16.9)
X^2^	14.517	12.875
P	<0.05	<0.05

Comparison of pain at the injection site between the two groups: The control group had more cases of severe pain than the research group (26 vs. 5), and the difference between the two groups was remarkable (P<0.05; Table-II).

**Table-II:** Comparison of pain at the injection site between the two groups.

Group pain	No pain	Mild pain	Moderate pain	Severe pain
Research group	0	99	26	5
Control group	0	47	57	26
u	4.094			
P	<0.05			

Comparison of bleeding severity at the 48^th^ h after subcutaneous injection between the two groups: The control group had more cases of severe bleeding than the research group (31 vs. 3), and the difference had statistical significance (P<0.05; Table-III).

**Table-III:** Comparison of bleeding degree between the two groups after subcutaneous injection.

Group	No bleeding	Mild pain	Moderate pain	Severe pain
Research group	94	21	12	3
Control group	62	16	21	31
u	3.029			
P	< 0.05			

## DISCUSSION

Low-molecular-weight heparin with strong anti-thrombus function is usually used for treating unstable angina pectoris, acute myocardial infarction and cerebral infarction. Subcutaneous injection of low-molecular-weight heparin can achieve an effective heparin concentration and 90% of bioavailability, which can fully reduce hypercoagulability and resist thrombogenesis. It has advantages of simple operation, good absorption, low anticoagulation and stable and lasting action; moreover detection of clotting mechanism is not needed.^10^,^11^ But in the injection process of low-molecular-weight heparin, incorrect injection method, improper injection sites and space between injection sites and incorrect compression strength and time may induce subcutaneous injection.^12^ Moreover most of patients are elderly.^13^ Elders may have heavy psychological burden because of the dissatisfaction on the society, family and medical care and fear of diseases and death, which can affect the treatment and recovery of patients. Moreover elders have high risks of bleeding because of aging of organs, decline of physiological function, poor vascular elasticity and decline of ability to resist external damages and cell repairing ability. Therefore much attention is needed regarding subcutaneous injection of low-molecular-weight heparin for elderly patients.

The traditional subcutaneous injection requires tightening of skin and a needle insertion angle between 30° and 40°. Skin tightening may make blood capillary easy to be punctured. Moreover if the needle insertion angle is small, drugs will be easily pushed into subcutaneous tissue to induce pain. If the angle is large, drugs will be pushed into muscular layer which has rich blood capillary; edema or induration may occur after blood capillary is punctured.^14^,^15^ In the controlled trial of left and right hands designed by Chen FJ^16^, hot compression on injection sites with hot towel before injection significantly relieved subcutaneous hemorrhage and pain, and effective message could achieve the same effect. It might be because that hot compression and message could accelerate blood circulation of local skin and reduce drug concentration. Punching up skin until injection ends can enlarge subcutaneous space and relax small blood vessels to benefit the diffusion and absorption of drugs and moreover can stop bleeding and prevent drug reflux. Vertical needle insertion and withdrawal can reduce bleeding and pain. Duan N found that vertically inserted needle was 5 to 10 mm deeper than obliquely inserted needle^17^, which meant the distance between drugs at injection site was far away from dermal tissue layer containing rich pain nerve.

The results of this study suggested that the modified injection method achieved good effect. Only five patients in the research group had severe pain, while the control group had 26 patients. Moreover the bleeding condition at the 24th h after injection indicated that 3 patients in the research group and 31 patients in the control group had severe bleeding, and there was a notable difference.

## CONCLUSION

Modified technique of subcutaneous injection of low-molecular-weight heparin can effectively reduce pain and subcutaneous ecchymosis, which is beneficial to the recovery of patients. But only 260 patients were enrolled in the study, and injection time could affect the bleeding rate; hence multi-center randomized controlled trials with large sample size are needed to supplement the results.

### Authors’ Contribution

**WZG:** Study design, data collection and analysis.

**WZG, YZ & JS:** Manuscript preparation, drafting and revising.

**WZG & YZ:** Review and final approval of manuscript.
